# Duloxetine in Psychiatric Disorders: Expansions Beyond Major Depression and Generalized Anxiety Disorder

**DOI:** 10.3389/fpsyt.2019.00772

**Published:** 2019-10-25

**Authors:** Maria Rosaria Anna Muscatello, Rocco A. Zoccali, Gianluca Pandolfo, Paolo Mangano, Simona Lorusso, Clemente Cedro, Fortunato Battaglia, Edoardo Spina, Antonio Bruno

**Affiliations:** ^1^Department of Biomedical and Dental Sciences and Morphofunctional Imaging, University of Messina, Italy; ^2^Department of Clinical and Experimental Medicine, University of Messina, Italy; ^3^Department of Clinical Neurosciences, Villa San Benedetto Menni, Italy; ^4^Department of Medical Sciences, Neurology and Psychiatry, Hackensack Meridian School of Medicine, Seton Hall University, United States

**Keywords:** duloxetine, psychiatric disorders, persistent depressive disorder, seasonal affective disorder, premenstrual dysphoric disorder, schizophrenia

## Abstract

**Background:** Duloxetine hydrochloride (DUL) is an antidepressant included in the pharmacological class of serotonin–norepinephrine reuptake inhibitors approved for the treatment of major depressive disorder, generalized anxiety disorder, diabetic peripheral neuropathic pain, fibromyalgia, and chronic musculoskeletal pain. The aim of this review was to elucidate current evidences on the use of DUL in the treatment of a variety of psychiatric disorders.

**Methods:** This systematic review was conducted according to PRISMA (Preferred Reporting Items for Systematic Reviews and Meta-Analyses) guidelines. PubMed database was searched from January 1, 2003, to September 30, 2018, using 11 key terms related to psychiatric disorders (“persistent depressive disorder,” “dysthymic disorder,” “bipolar disorder,” “seasonal affective disorder,” “obsessive-compulsive disorder,” “social phobia,” “panic disorder,” “posttraumatic stress disorder,” “schizophrenia,” “eating disorders,” “sexual disorders,” “personality disorders”) and one key term related to duloxetine (“duloxetine hydrochloride”). Article titles and abstracts were scanned to determine relevance to the topic. For additional studies, the authors also examined the reference lists of several of the included papers.

**Results:** Duloxetine may be an effective treatment for mood spectrum disorders, panic disorder, several symptom clusters of borderline personality, and as add-on drug in schizophrenia. Modest or conflicting results have been found for the efficacy of duloxetine in obsessive–compulsive disorder, posttraumatic stress disorder, eating, and sexual disorders.

**Conclusion:** Major limitations of the reviewed studies were short trial duration, small sample sizes, and the lack of control groups. Defining the potential role of DUL in the treatment of psychiatric disorders other than major depressive disorder and generalized anxiety disorder needs further randomized, placebo-controlled studies.

## Introduction

Duloxetine hydrochloride (DUL) [LY248686; (+)-N-methyl- 3-(1-naphthalenyloxy)-2 thiophenepropanamine] is an antidepressant included in the pharmacological class of serotonin (5-HT)-norepinephrine (NE) reuptake inhibitors (SNRIs), a class that also comprises venlafaxine, desvenlafaxine, milnacipran, and levomilnacipran. Initially approved for the treatment of major depressive disorder (MDD) by the US Food and Drug Administration (FDA) in 2004 and, subsequently, by the Committee for Medicinal Products for Human Use in Europe, actually the drug is approved in a number of countries for the treatment of generalized anxiety disorder (GAD), diabetic peripheral neuropathic pain, fibromyalgia (FM), chronic musculoskeletal pain, and, in Europe, also for treating stress urinary incontinence (1). In addition, it has been proposed for patients with chemotherapy-induced neuropathies (2), and for chronic postsurgical pain (3).

The pharmacological profile of DUL has been characterized by a series of *in vitro* and *in vivo* experimental studies. In vitro, DUL preferentially inhibits 5-HT reuptake more than NE reuptake (4, 5); DUL binding capacity to the human 5-HT and NE transporters is 100 times more potent than venlafaxine, whereas its affinity for NE reuptake inhibition is lower than those of milnacipran and levomilnacipran (6, 7). DUL lacks affinity for monoamine receptors within the central nervous system and shows few effects on the histaminic H1, muscarinic, α1-adrenergic, and opioid receptors (8). Chronic duloxetine treatment exerts a long-term modulatory effect on 5-HT and NE pathways, demonstrating no effect on basal 5-HT, NE, or dopamine levels in the cerebral cortex, a moderate effect on 5-HT and NE release in the hippocampus, and a substantial desensitization of terminal α2-heteroreceptors but not 5-HT1B receptors (9). Based on these evidences, it can be hypothesized that chronic DUL treatment involves adaptive changes of autoreceptor functions, similarly to what happens in chronic treatment with selective serotonin reuptake inhibitors (SSRIs) and noradrenaline reuptake inhibitors (NRIs), which fail to modify 5-HT and NE levels, respectively (10).

Other potential mechanisms linked to the antidepressant activity of DUL involve its effects on neurotrophin levels and neuronal plasticity. As well as other antidepressants, such as SSRIs and NRIs, chronic but not acute DUL treatment increases cortical and hippocampal expression of the mature form of brain-derived neurotropic factor (BDNF) that promotes neuronal survival and differentiation, differently from the precursor of BDNF (pro-BDNF), which increases programmed neuronal death (11, 12). Beyond its effect on BDNF, chronic DUL treatment augments the expression of the growth factor and immediate early gene activity-regulated cytoskeleton-associated protein (Arc) that has a substantial role in neural plasticity (13).

Since both 5-HT and NE monoamines are not only involved in the pathophysiology of depression, but also modulate ascending spinal nociceptive neurotransmission *via* the descending inhibitory pain pathway (14), DUL shows analgesic properties in conditions of chronic pain (15). In addition, the inhibition of 5-HT and NE reuptake induced in the sacral spinal cord motor neurons that innervate the striated muscle of the urethral sphincter (16) makes DUL also effective in the treatment of stress urinary incontinence.

In summary, due to its clinical profile and mechanism of action, DUL may be a valuable option to treat disorders other than MDD and GAD. Therefore, the aim of this review was to elucidate current evidence on the use of DUL for the treatment of a variety of psychiatric disorders.

## Pharmacokinetics and Drug Interactions of Duloxetine

After oral administration, DUL reaches the maximum concentration in plasma (Cmax) in approximately 6hours. Concomitant administration with a meal increases the time to peak absorption by 6 to 10 hours and decreases the area under the concentration-versus-time curve (AUC) by 10% (17). The estimated volume of distribution is 1,640 L, bioavailability about 50%, and protein binding (mainly to albumin or α1-acidglycoprotein) up to 90% (17). Elimination of DUL (half-life of about 12 hours) occurs by hepatic biotransformation, *via* cytochrome P-450 (CYP) 1A2 and 2D6 isoforms, and by renal (70%) and fecal (20%) excretion. To date, none of the proposed major metabolites (any metabolite constituting >1% of the total) has been shown pharmacologically active (18). Since DUL is extensively metabolized by the liver, any degree of hepatic insufficiency is a contraindication to treatment; in hepatopathic patients, after a single dose of 20 mg, mean plasma clearance was significantly reduced, AUC increased fivefold, and the half-life was threefold longer of that observed in patients without hepatic dysfunctions. DUL may also exacerbate preexisting chronic liver disease and interact with alcohol, potentially resulting in liver injury; thus, it should not be prescribed in these cases. Similarly, since renal excretion has a major role in the catabolism of DUL, subjects with a creatinine clearance of <30 mL/min and patients affected by moderate or severe renal impairment should be prescribed adjusted dosages of DUL and should be monitored closely during treatment. Drug interactions are possible: DUL has been shown to be both a substrate and a moderate inhibitor of CYP2D6, and therefore it can compete for the same isoenzymes with other substrates, such as tricyclic antidepressants, phenothiazines, and type 1C antiarrhythmics (19). In a similar way to other agents in the SNRI class, DUL may be involved in pharmacodynamic drug interactions; particularly, the combination of the drug with monoamine oxidase inhibitors is contraindicated, due to the possible development of a serotonin syndrome, a potentially life-threatening adverse event.

## Tolerability and Safety Issues

The drug is generally safe and well tolerated across all approved indications in adults at doses ranging from 60 to 120 mg/day, although potential therapeutic benefits of high doses of DUL are associated with emergent side effects, without significant symptoms reduction and final remission rates (20). The most commonly reported adverse reactions (≥5% and at least twice the incidence of placebo patients) were nausea, dry mouth, somnolence, constipation, decreased appetite, and hyperhidrosis, which occurred mainly in the early stages of the assumption and disappeared after the first weeks of treatment (21). In the longer-term therapy (for at least 6 months to 1 year), frequent treatment-emergent adverse events observed in adult patients were palpitations, blurred vision, vertigo, weight gain/loss, chills/rigors, and pruritus (22). Regarding cardiovascular safety, a pooled analysis of clinical trials on MDD showed that DUL had modest effects on heart rate and blood pressure and no clinically meaningful effect on electrocardiogram (ECG) profiles; the cardiovascular effects of DUL were comparable with other antidepressants (23). However, it should be stressed that, in most studies, patients with unstable cardiovascular disease or preexisting ECG abnormalities were excluded, whereas the selected patients with cardiovascular disease had to be clinically stable and under treatment.

Regarding suicidality, a meta-analysis of differences in incidence and outcomes of suicidal behaviors during randomized trials of DUL versus placebo found no evidence of increase or decrease in risk of suicide-related events with DUL treatment (24). Whether antidepressant drugs with similar or different pharmacological profile can increase the risk of suicidality and aggressive behaviors is still a matter of debate, and discussing possible relationships among suicidality, antidepressant treatment, and MDD goes far beyond the aim of our review. However, based on a pooled analysis of trials of nine different antidepressants, which showed an increase in the suicidal ideation and behavior in children and adolescents with MDD for almost all drugs, the European Medicines Agency and the FDA required a black box warning about the use of DUL in children, adolescents, and young adults (25). Suicidal risk seems to be higher at treatment beginning, during dose-tapering phases, and after discontinuation of the drug.

Safety in overdose is one of the essential parameters that should guide antidepressants prescription, especially when considering that 50% of suicide attempts consist of drug overdose, and nearly 17% are made with the prescribed antidepressant (26). Overall, DUL is considered to be relatively safe in the case of overdose, but the possibility of fatal outcomes increases with concomitant assumption of multiple drugs. Symptoms of DUL overdose are somnolence, hypotension or hypertension, vomiting, tachycardia, syncope, serotonin syndrome, seizures, and coma. There is no specific antidote for DUL; thus, general measures for intoxication are recommended. Beyond monitoring of vital signs, gastric lavage and activated charcoal for limiting the absorption are suitable if performed soon after ingestion (25).

As also reported for other antidepressants, withdrawal reactions and discontinuation-emergent adverse events may occur after gradual or abrupt suspensions of DUL treatment; the rates of withdrawal symptoms, mainly nausea, ranged from 6% to 55% (27). This possibility should not be underestimated, and in the case of DUL, this issue is particularly important, since this agent is frequently prescribed in general care settings, for pain disorders, FM, and stress urinary incontinence.

## Methods

### Research Strategy

This systematic review was conducted according to PRISMA (Preferred Reporting Items for Systematic Reviews and Meta-Analyses) guidelines (28). PubMed database was searched from January 1, 2003, to September 30, 2018, using 11 key terms related to psychiatric disorders (“persistent depressive disorder,” “dysthymic disorder,” “bipolar disorder,” “seasonal affective disorder,” “obsessive-compulsive disorder,” “social phobia,” “panic disorder,” “posttraumatic stress disorder,” “schizophrenia,” “eating disorders,” “sexual disorders,” “personality disorders”) and one key term related to duloxetine (“duloxetine hydrochloride”). The electronic search strategy used for PubMed is described in **Table 1**.

**Table 1:** List of search terms entered into the PubMed search engines for identification of the studies within the scope of this systematic review.

Number	Search terms
1	PERSISTENT DEPRESSIVE DISORDER
2	DYSTHYMIC DISORDER [all fields]
3	BIPOLAR DISORDER [all fields]
4	SEASONAL AFFECTIVE DISORDER [all fields]
5	OBSESSIVE-COMPULSIVE DISORDER [all fields]
6	SOCIAL PHOBIA [all fields]
7	PANIC DISORDER [all fields]
8	POSTTRAUMATIC STRESS DISORDER [all fields]
9	SCHIZOPHRENIA [all fields]
10	EATING DISORDERS [all fields]
11	SEXUAL DISORDERS [all fields]
12	PERSONALITY DISORDERS [all fields]
13	DULOXETINE HYDROCLORIDE [all fields]
14	1 OR 2 OR 3 OR 4 OR 5 OR 6 OR 7 OR 8 OR 9 OR 10 OR 11 OR 12
15	14 AND 13
16	English [language]
17	2003/01/01 to 2018/09/30 [publication date]

Articles have been selected by title and abstract; the entire article was read if title/abstract indicated that duloxetine was used for treating psychiatric disorders other than MDD and GAD, and if the article potentially met the inclusion criteria. References of the selected articles were also examined in order to identify additional studies meeting the inclusion criteria.

### Study Selection

Articles were included in the review according to the following inclusion criteria: English language, publication in peer reviewed journals, quantitative information on the use of duloxetine in psychiatric disorders other than MDD and GAD, and year of publication at least 2003. Articles were excluded by title, abstract, or full text for MDD and GAD diagnoses, also in comorbidity, and for irrelevance to the topic in question. Further exclusion criteria were review articles, editorial comments, case reports/series, and animal model studies.

### Data Extraction

Two authors (PM, SL) performed the initial search, independently reviewed and selected the references based on the inclusion and exclusion criteria, and clarified any disputes in the presence of a third expert reviewer (MRAM). The results were subsequently reevaluated by the auditors, and the salient results were shown. After having discarded duplicate articles, data derived from our research of articles included study author names, publication dates, study designs (i.e., open-label uncontrolled and randomized controlled trial), sample (case and control group), duloxetine regimen, active comparator regimen, primary outcome measures, and main efficacy results (response and remission rates, mean changes in outcome measures).

Principal outcome of interest included studies about duloxetine efficacy on psychopathological symptoms.

## Results

### Study Characteristics

**Figure 1:**
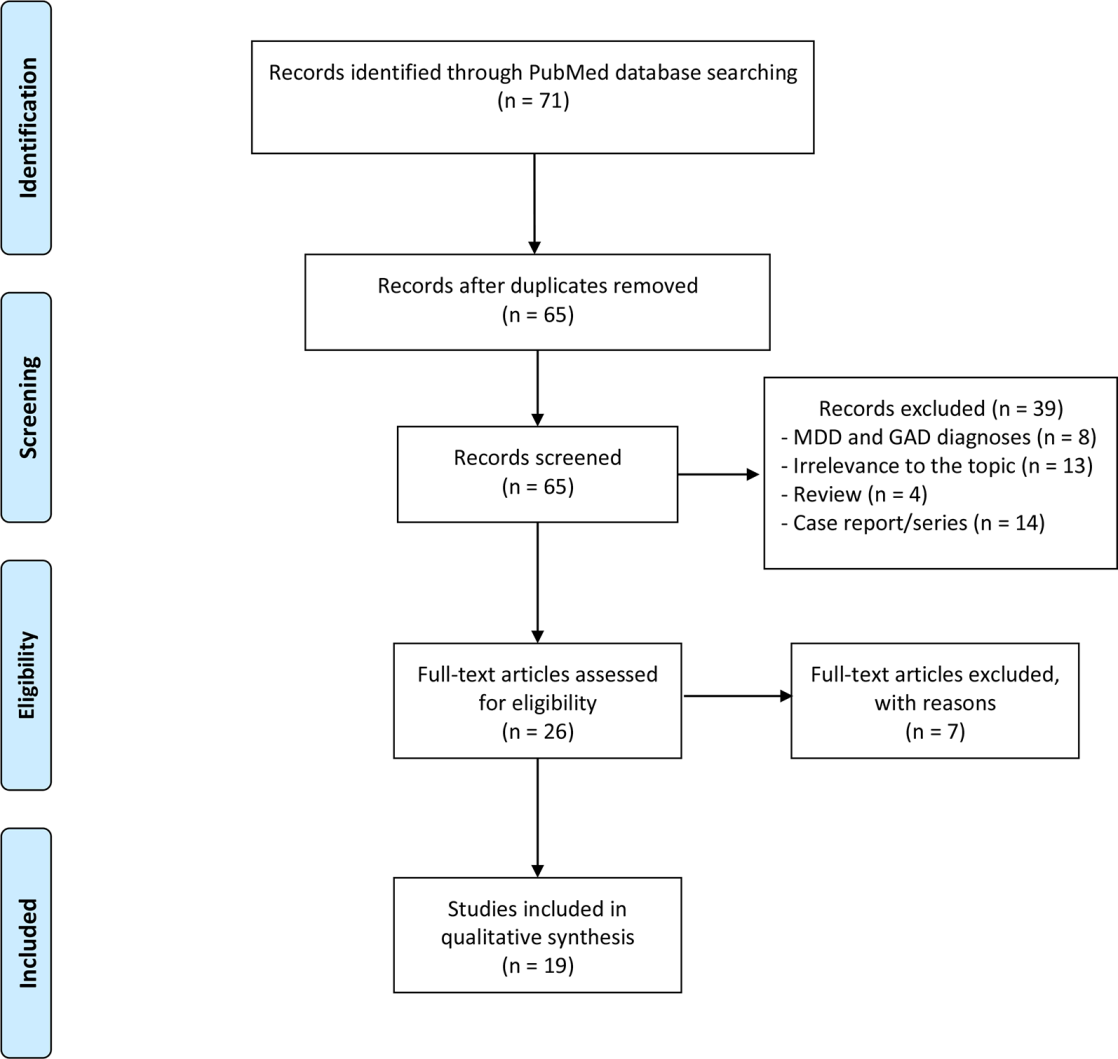
Flow diagram of the literature selection process.

**Figure 1** summarizes the flowchart of articles selected for the review. The search of PubMed database provided a total of 71 citations; no additional studies meeting inclusion criteria were identified by checking the reference list of the selected papers. After adjusting for duplicates, 65 records were screened. Of these, 8 studies were excluded because these were related to MDD and GAD diagnoses, 13 were irrelevant to the topic, 4 were reviews, and 14 were case report/series.

After the screening, a total of 19 studies assessing the use of duloxetine in psychiatric disorders other than MDD and GAD met the inclusion criteria and were included in the systematic review (**Tables 2** and **3**); in particular, 7 articles focused on other mood disorders, and 12 studies evaluated duloxetine efficacy in other psychiatric disorders (obsessive–compulsive disorder [OCD]/panic disorder (PD)/posttraumatic stress disorder (PTSD)/eating disorders/schizophrenia/premature ejaculation [PE]/attention-deficit/hyperactivity disorder [ADHD]/borderline personality disorder [BPD]).

### Use of Duloxetine in Other Mood Disorders

The efficacy of DUL in patients with mood disorders other than MDD has been evaluated in seven clinical trials (**Table 2**).

**Table 2:** Published efficacy trials of duloxetine in other mood disorders.

Authors/year of publication	Study design	Trial duration	Number of patients	Duloxetine regimen	Active comparator regimen	Primary outcome measure	Main efficacy results
Persistent depressive disorder							
Koran et al., 2007 (29)	Open-label, uncontrolled	12 weeks	24	60–120 mg/day	NA	IDS-C	IDS-C (ITT): response rate = 83%; remission rate = 79%
Kerner et al., 2014 (30)	Open-label, uncontrolled	12 weeks	30	20–120 mg/day	NA	HDRS	HDRS (ITT): response rate = 53.3%; remission rate (ITT) = 33.3%; daily doses above 60 mg were associated with greater improvement and well tolerated
Hellerstein et al., 2012 (31)	Randomized, double-blind, placebo-controlled	10 weeks	65	30–120 mg/day	NA/Pl	HDRS	HDRS: response rate DUL vs. Pl = 65.5% vs. 25.0% (p = 0.003); remission rate DUL vs. Pl = 55.2% vs. 14.3% (p = 0.002)
Hellerstein et al., 2017 (32)	Open-label, observational	12 weeks	19 D24 Pl → D	30–120 mg/day	NA	HDRS	Patients continuing DUL: response criteria = 84%, remission criteria = 63%.Patients initially Pl: response criteria = 83%, remission criteria = 62%
Seasonal affective disorder							
Pjirek et al., 2008 (33)	Open-label, uncontrolled	8 weeks	26	60–120 mg/day	NA	SIGH-SAD	SIGH-SAD (ITT): response rate = 80.8%; remission rate = 76.9%
Premenstrual dysphoric disorder							
Mazza et al., 2008 (34)	Open-label, uncontrolled	Two menstrual cycles	55	60 mg/day	NA	VAS-Mood	VAS-Mood: response rate = 78%
Ramos et al., 2009 (35)	Single-blind, uncontrolled	Three menstrual cycles	20	60 mg/day	NA/Pl	DRSP	DRSP (ITT): response rate = 65%

DUL, duloxetine; NA, not available; Pl, placebo; IDS-C, Inventory for Depressive Symptomatology; HDRS, Hamilton Depression Rating Scale; SIGH-SAD, Structured Interview Guide for the Hamilton Rating Scale–SAD version; VAS-Mood, visual analog scale–mood; DRSP, Daily Record of Severity of Problems; ITT, intent-to-treat.

**Table 3:** Published efficacy trials of duloxetine in other psychiatric disorders.

Authors/year of publication	Study design	Trial duration	Number of patients	Duloxetine regimen	Active comparator regimen	Primary outcome measure	Main efficacy results
Obsessive–compulsive disorder							
Dougherty et al., 2015 (36)	Open-label, uncontrolled	17 weeks	20	60–120 mg/day	NA	Y-BOCS	Y-BOCS (ITT): full responders = 35%; nonresponders = 45%
Mowla et al., 2016 (37)	Randomized, double-blind	8 weeks	46	20–60 mg/day	Sertraline 50-200 mg/day	Y-BOCS	Y-BOCS: DUL response rate = 62.5%; S response rate = 62.5%
Panic disorder							
Simon et al., 2009 (38)	Open-label, uncontrolled	8 weeks	15	30–120 mg/day	NA	PDSS	PDSS: mean (SD) Baseline vs. endpoint = 14.2 (4.2) vs. 9.13 (5.2) (*P* = 0.001)
Posttraumatic stress disorder							
Villarreal et al., 2010 (39)	Open-label, uncontrolled	12 weeks	20	30–120 mg/day	NA	CAPS	CAPS (ITT): response rate = 45%; remission rate = 5%
Eating Disorders							
Leombruni et al., 2009 (40)	Open-label, uncontrolled	12 weeks	45	60–120 mg/day	NA	BES	BES (ITT): mean (SD) baseline vs. endpoint = 28.8 (8.2) vs. 22.9 (11.0) (*P* = 0.001)
Guerdjikova et al., 2012 (41)	Randomized, double-blind, placebo-controlled	12 weeks	40	30–120 mg/day	NA/Pl	Binge day frequency	Binge day frequency: remission rate DUL vs. Pl = 56% vs. 30% (*P* = 0.09)
Schizophrenia							
Micò et al., 2011 (42)	Randomized, double-blind, placebo-controlled augmentation study (clozapine)	16 weeks	40	60 mg/day	NA/Pl	PANSS	PANSS change (ITT): T mean (SD) DUL vs. Pl = −15.9 (14.9) vs. 0.8 (3.9) (*P* < 0.0001); N mean (SD) DUL vs. Pl = −4.4 (3.1) vs. 0.7 (1.9) (*P* < 0.0001); GP mean (SD) DUL vs. Pl = −10.4 (11.7) vs. 0.3 (2.6) (*P* < 0.0001)
Nikbakhat et al., 2016 (43)	Randomized, double-blind, placebo-controlled augmentation study (risperidone)	8 weeks	64	60 mg/day	NA/Pl	PANSS	PANSS scores: T mean (SD) DUL vs. Pl = 24.6 (7.2) vs. 19.3 (3.4) (P = 0.001); N mean (SD) DUL vs. Pl = 6.1 (2.0) vs. 4.1 (1.1) (*P* = 0.001); GP mean (SD) DUL vs. Pl = 9.9 (2.9) vs. 7.9 (1.2) (*P* = 0.001)
Premature ejaculation							
Athanasios et al., 2007 (44)	Randomized, double-blind, placebo-controlled	12 weeks	20	80 mg/day	NA/Pl	IELT	IELT: mean time (SD) DUL vs. placebo = 129.34 (67.5) vs. 38.61 (16.9) (*P* < 0.001)
Ozcan et al., 2015 (45)	Randomized, double-blind	4 weeks	80	40 mg/day	Paroxetine 20 mg/day	IELT	IELT: increasing rate DUL vs. P = 117% vs. 126% (*P* > 0.05)
Attention-deficit/hyperactivity disorder							
Bilodeau et al., 2014 (46)	Randomized, double-blind, placebo-controlled	6 weeks	30	60 mg/day	NA/Pl	CAARS-Inv : SV	CAARS-Inv : SV: mean DUL vs. Pl = 25.67 vs. 31.33 (*P* > 0.05)
Borderline personality disorder							
Bellino et al., 2010 (47)	Open-label, uncontrolled	12 weeks	18	60 mg/day	NA	BPDSI	BPDSI: significant improvement in total score (*P* = 0.001), “Impulsivity” (*P* = 0.028), “outbursts of anger” (*P* = 0.0005), “affective instability” (*P* = 0.001)

DUL, duloxetine; S, sertraline; P, paroxetine; Pl, placebo; NA, not available; Y-BOCS, Yale–Brown Obsessive Compulsive Scale; PDSS, Panic Disorder Severity Scale; CAPS, Clinician-Administered PTSD Scale; PCL-C, PTSD Checklist; BES, Binge Eating Scale; PANSS (T, P, N, GP): Positive and Negative Syndrome Scale (total score, positive, negative, and general psychopathology subscales); IELT, intravaginal ejaculation latency time; CAARS-Inv : SV, Conners’ Adult ADHD Rating Scale-Investigator Report: Screening Version; BPDSI, Borderline Personality Disorder Severity Index; ITT, intent-to-treat.

The efficacy and safety of DUL on persistent depressive disorder (PDD), or dysthymia, or dysthymic disorder (DD) has been evaluated in four studies: two open-label trials and one randomized, controlled trial followed by an open extension study. The first, open-label, 12-week study (29), examined 24 adults with *Diagnostic and Statistical Manual of Mental Disorders, Fourth Edition* dysthymia alone or with concurrent major depression (“double depression”) who received duloxetine 60 mg/day for 6 weeks, increased to 120 mg/day for the remainder of the 12-week trial for those who showed a partial treatment response. Outcome measure was the clinician-rated Inventory for Depressive Symptomatology (IDS-C), whose mean scores significantly decreased from baseline to endpoint under DUL treatment. Among study completers, the IDS-C response rate was 89% (17/19), and the remission rate 84% (16/19); five subjects (21%) dropped out for unwanted side effects.

A more recent open-label, 12-week trial (30) evaluated the efficacy of duloxetine for DD in 30 older adults (mean age = 70.7 years; completers = 19). Duloxetine was prescribed at flexible doses, starting from 20 mg/day (first week) up to 120 mg daily. Depressive symptoms improved with duloxetine as assessed by the 24-item Hamilton Depression Rating Scale (HDRS) and the more specific Cornell Dysthymia Rating Scale (CDRS); response (≥50% decrease in HDRS scores with a Clinical Global Impression [CGI] score of “much improved”) rate was 53%, and remission rate 33.3%, with remission defined as a final HDRS score ≤6. Out of the total number of 11 dropouts during the study, 16.7% were due to adverse effects (dry mouth, weakness, sexual dysfunction, and constipation).

The double-blind, randomized 10-week trial examined the efficacy of DUL (dosage range, 30–120 mg/day) on DD or depression not otherwise specified in a sample of 57 patients (31). After 10 weeks, duloxetine-treated subjects had significantly lower scores than placebo-treated subjects on the primary outcome measure, the 24-item HDRS (*P* = 0.003), and on secondary measures (CDRS, CGI), whereas no effect of DUL treatment was seen on Beck Depression Inventory (BDI), Global Assessment of Functioning, and Social Adjustment Scale (SAS). The response rate was 65.5% for DUL versus 25.0% for placebo (χ^2^_1_ = 9.43, *P* = 0.003), and the remission rate was 55.2% for DUL versus 14.3% for placebo (χ^2^_1_ = 10.46, *P* = 0.002). On the basis of the observed residual deficits in functioning even after clinical symptoms remission, the authors designed an additional 12-week continuation phase of the study, in which participants (DUL group n = 19) were provided with open DUL treatment, including those initially assigned to placebo (n = 24) (32). Sustained symptoms improvement was seen in patients continuing DUL, with 84% meeting response and 63% remission criteria at endpoint (week 22). Patients switching from placebo to DUL showed similarly high levels of response (83%) and remission (62%) at endpoint. Nevertheless, in the longer term, DUL-continuation patients improved only modestly on social and cognitive functioning, with 66.7% of patients persisting in the impaired range of functioning according to the SAS.

Only one open-label study examined DUL efficacy and tolerability in 26 subjects affected by seasonal affective disorder (SAD) (33). All participants were assessed by the Structured Interview Guide for the Hamilton Rating Scale–SAD version (SIGH-SAD), the Clinical Global Impression of Severity (CGI-S), the Clinical Global Impression of Improvement (CGI-I), the CGI Efficacy Index, the Social Adaptation Self-evaluation Scale, and the Sheehan Disability Scale (SDS) and received 60 to 120 mg/day of DUL for 8weeks. Primary outcome variables were SIGH-SAD total score; response was defined as a reduction of SIGH-SAD total score of more than 50% from baseline score, and remission was a SIGH-SAD total score of 7 or lower. Results showed that DUL treatment (21 patients were treated with 60 mg, and 5 received 120 mg) had a significant effect on SIGH-SAD total score (*F*_1.74_ = 55.20, *P* < 0.001). At endpoint, 21 patients (80.8%) had responded to DUL treatment, and 20 patients (76.9%) had experienced remission according to the above defined criteria. Patients showed marked improvement on secondary outcome measures of social functioning. Fifty-five treatment-emergent adverse events were reported during the trial, 26 (47.3%) of which were classified as mild (palpitations, tremors, sedation, loss of weight), 21 (38.2%) as moderate (headache, insomnia, inner tension), and 8 (14.5%) as severe (dry mouth, hyperhidrosis, constipation).

Two studies have evaluated DUL efficacy and safety on premenstrual dysphoric disorder (PMDD). The first was an open-label, fixed-dose (60 mg/day) study (34) on a sample of 55 subjects (50 completers). All subjects were assessed by a visual analog scale (VAS) recording 11 mood symptoms (primary outcome measure), the HDRS, the Hamilton Anxiety Rating Scale (HARS), the Zung Self-rating Scale for Depression, and the CGI-S. DUL, administered for two menstrual cycles, was effective during the first cycle in reducing mood symptoms, with 39 subjects (78%) meeting criteria for treatment response, defined as at least 50% reduction in luteal VAS mood score from baseline to endpoint. A concomitant improvement in anxiety symptoms and in general functioning was seen. Adverse events during the treatment phase were nausea (three subjects [6%]), insomnia (two subjects [4%]), and poor appetite (two subjects [4%]). A single-blind, uncontrolled, fixed-dose (60 mg/day) trial evaluated the effect of DUL on 20 patients (15 completers) with PMDD (35). All patients were rated with the short form of Daily Record of Severity of Problems (DRSP), a 14-item patient-rated scale that incorporates all symptoms listed in the *Diagnostic and Statistical Manual of Mental Disorders, Fourth Edition, Text Revision* diagnostic criteria for PMDD, the 17-item HDRS, CGI-S, CGI-I, SDS, and the short form of the Quality of Life Enjoyment and Satisfaction Questionnaire (Q-LES-Q). DUL or placebo (for one treatment cycle) was administrated for up to three menstrual cycles. The response rate to treatment (≥50% reduction in total DRSP scores from baseline to endpoint) was 65% (intention-to-treat [ITT] population 13/20); the patients also experienced a significant reduction of functional impairment associated with premenstrual symptoms. Common and generally transient side effects at the first treatment cycle were gastrointestinal disturbances and decreased appetite, whereas headaches, decreased libido, insomnia, and sweating persisted throughout the treatment cycles (three patients dropped out for persistent, although not serious, adverse effects).

### Use of Duloxetine in Other Psychiatric Disorders

The efficacy of DUL in patients with psychiatric disorders other than mood disorders has been evaluated in 12 clinical trials (**Table 3**).

#### Obsessive–Compulsive Disorder

To date, the use of DUL in the treatment of patients with OCD has been assessed in one open-label study and one double-blind, controlled augmentation trial. The 17-week, open-label trial by Dougherty et al. (36) evaluated the efficacy and safety of DUL on OCD symptom severity as assessed by the Yale–Brown Obsessive Compulsive Scale (YBOCS) and CGI (primary outcome measures); psychometric examination also included BDI, Beck Anxiety Scale, and the Q-LES-Q (secondary and tertiary outcome measures). The total sample was formed by 20 OCD patients (12 completers); results evidenced that, of the 12 completers, 7 (58.3%) were full responders and 3 (25%) were nonresponders according to primary outcome measures; DUL at the dose range of 60 to 120 mg/day was effective for reducing YBOCS total score (mean ± SD = 28.33 ± 4.66 at baseline, 18.5 ± 7.98 at endpoint; *P* < 0.001) and CGI score (mean ± SD = 4.00 ± 0 at baseline, 2.17 ± 0.72 at endpoint; *P* < 0.001). Five participants out of 12 (40%) discontinued the study because of adverse events, and two patients required a reduction in dosage due to adverse events. The most common unwanted effects were nausea (50% of subjects), fatigue (41.2%), sexual dysfunction (23.1%), and headache (11.1%); no serious adverse events occurred.

The 8-week randomized controlled, double-blind study (37) assessed the efficacy of adjunct DUL or sertraline in patients with resistant OCD treated with SSRIs or fluvoxamine. Forty-six OCD patients who had failed an average of two SSRI trials of adequate dose and duration before the start of the study were randomly allocated to receive, under a double-blind condition, DUL (dose range = 20–60 mg/day; mean dosage = 44.4 mg/day; n = 24) or sertraline (dose range = 50–200 mg/day; mean dose = 123.8 mg/day; n = 22). The primary outcome measure was the YBOCS; the efficacy index of CGI was used at the end of the study. At endpoint, both DUL and sertraline were effective in reducing OCD symptoms, as assessed by YBOCS mean total score reduction (33.0% for DUL and 34.5% for sertraline); 15 DUL-treated patients and 13 sertraline-assuming subjects were considered responders. Six patients in the DUL group and five patients in the sertraline group dropped out because of unwanted side effects; the more common were gastrointestinal disturbances, followed by headache and sexual disturbances.

#### Panic Disorder

A single, 8-week, open-label, flexible-dose study (38) has evaluated the efficacy of DUL (dose range = 30–120 mg/day; mean ± SD dose at endpoint = 94 ± 25 mg/day) in 15 patients (12 completers) with PD. Primary outcome measure was the Panic Disorder Severity Scale; secondary measures included panic attack frequency as measured by the Panic Attack Scale and the CGI-S. Results suggested that DUL was associated with a significant improvement in symptom severity (all primary and secondary measures = *P* < 0.01), with eight patients not experiencing full panic attacks in the past 2 weeks and four achieving full remission. The most common side effects were nausea, sedation, and sexual dysfunction. DUL was generally well tolerated, with only two patients discontinuing the drug for side effects both in the first week on medication (one due to insomnia and loss of appetite, one for worsened depression).

#### Posttraumatic Stress Disorder

To date, the efficacy and tolerability of DUL in PTSD have been evaluated in a 12-week open-label trial on a sample of 20 (15 completers) military veterans affected by PTSD (39). Primary efficacy measure was the Clinician-Administered PTSD Scale (CAPS), assessing the severity of PTSD symptomatology, whereas secondary measures included the HDRS. Duloxetine (mean daily dose = 81 mg/day) was effective in reducing PTSD and depressive symptoms, as assessed by changes in primary and secondary outcome measures, with nine participants (45%) classified as responders, defined by 20% or greater improvement on CAPS total score. Of the total number of five subjects who dropped out from the trial, three were due to side effects (constipation, diarrhea, and nausea were the most common); two subjects developed tachycardia, and one withdrew from the trial for this problem.

#### Eating Disorders

The efficacy of DUL on binge-eating disorders (BEDs) has been evaluated in trials, one open-label and one placebo-controlled. A preliminary, 12-week, open-label study (40) tested the efficacy of flexible doses of DUL (dose range = 60–120 mg/day) in a sample of 45 obese outpatients who satisfied the full criteria for BED (n = 22) and in subjects (n = 23) with subthreshold binge eating, characterized by high eating impulsivity. Number of binge-eating episodes per week and scores on the Binge Eating Scale (BES) were the primary outcome measures; effect on weight, body mass index (BMI), depressive symptoms (BDI score), and CGI-S were the secondary outcome measures. The 31 completers reported a significant reduction in BES scores, number of binges, weight, BMI, and BDI scores, as well as in CGI-S scores; 14 subjects dropped out for reasons unrelated to side effects. DUL was generally tolerated: most frequent side effects were nausea and insomnia. A double-blind study of duloxetine versus placebo was carried out in 2011, involving 40 subjects with BED comorbid with depressive disorders (41). The administration of DUL started from the dose of 30 mg/day for the first week, to 60 mg/day for the second week, possibly 90 mg/day from the fourth week, and 120 mg/day from the sixth week, when positive effects were not reached, and DUL was well tolerated. Endpoints were the intensity and the number of episodes of binge eating, BMI, and depressive symptoms. After the end of the trial, DUL was proven to be effective in ameliorating the selected parameters.

#### Schizophrenia

The first report of DUL add-on in schizophrenia was a double-blind, 16-week, randomized trial on a sample of 40 (33 completers) schizophrenia patients classified as partial responders to clozapine according to a score ≥25 on the Brief Psychiatric Rating Scale (BPRS). DUL, at the dose of 60 mg/day, was proven effective in improving negative and general psychopathology symptoms, as documented by the reduction of negative subscale scores of the Positive and Negative Syndrome Scale (PANSS) and BPRS scores, respectively. Cognitive functioning, as measured by Stroop test, verbal fluency, and Wisconsin Card Sorting Test, did not change during DUL treatment. Combined therapy of DUL and clozapine was generally well tolerated, with gastrointestinal symptoms and headache as the most commonly reported side effects; 15% of the dropouts were in the treated group and 20% in the control group (42). More recently, an 8-week, randomized, double-blind, placebo-controlled, parallel-group trial aimed at evaluating DUL 60 mg/day added to risperidone (dose range = 4–6 mg/day) for treating negative symptoms in 64 schizophrenia patients (43). DUL was effective in improving negative and general psychopathology symptoms of schizophrenia, as shown by reductions in PANSS total and subscales scores. Adverse events did not differ between DUL and placebo groups.

#### Premature Ejaculation

Two studies have evaluated DUL efficacy and safety in the treatment of PE. In a 12-week study (44), 20 subjects who had been diagnosed with PE were randomly allocated to receive DUL (up to 80 mg/day; n = 10 subjects) or placebo (n = 10 subjects). The intravaginal ejaculation latency time (IELT), defined as the duration between vaginal intromission and ejaculation, was the outcome measure. All 20 participants completed the treatment. At endpoint, the IELT significantly increased in DUL group but not in placebo group (mean time ± SD DUL vs. placebo = 129.34 ± 67.58 vs. 38.61 ± 16.99; *P*<0.001). Common side effects were nausea and dry mouth for three patients in the active group and excessive sweating for one patient from placebo group; no patient dropped out from each treatment group because of adverse events. A more recent 4-week study (45) has examined the efficacy and tolerability of DUL versus paroxetine in PE in a sample of 80 patients randomly distributed to receive DUL 40 mg/day (N = 40; mean ± SD baseline IELT = 55 ± 5.8 s) or paroxetine 20 mg/day (N = 40; mean ± SD baseline IELT = 54.3 ± 5.9 s). The International Index of Erectile Function Questionnaire, IELT, and Premature Ejaculation Profile, assessing personal and interpersonal distress related to ejaculation, perceived control over ejaculation, and satisfaction with sexual intercourse, were recorded before and after treatment. Both DUL and paroxetine were effective in increasing IELT from baseline to endpoint (117% in the DUL, *P* < 0.001, and 126% in the paroxetine group, *P* < 0.001; no statistical differences between the two groups in terms of IELT increase were found). Both treatments were also effective in decreasing mean scores for personal distress and interpersonal difficulty related to ejaculation (*P* < 0.001). Both treatments were well tolerated, and none of the patients withdrew from the study; the most common side effects were nausea (N = 10) and headache (N = 5) in DUL-treated patients, sedation (N = 10), nausea (N = 10), and asthenia (N = 5) in the paroxetine group.

#### Adult Attention-Deficit/Hyperactivity Disorder

A 6-week, randomized, double-blind, placebo-controlled trial (46) has explored the efficacy and safety of DUL at the daily dose of 60 mg in a sample of 30 adults (24 completers) with ADHD. Primary efficacy measures were the Conners’ Adult ADHD Rating Scale–Investigator Report: Screening Version (CAARS-Inv : SV), the CGI-S, and the CGI-I. At the end of the trial, no group differences were found on CAARS-Inv : SV, whereas significant improvements were seen in DUL group in CGI-S (3.00 vs. 4.07 for placebo, *P* < 0.001) and CGI-I (2.89 vs. 4.00 at week 6, *P* < 0.001) scores. Main side effects of DUL treatment were nausea, xerostomia, headache, increased anxiety, constipation, and blurred vision; during the first week of treatment, there were six dropouts in the DUL group due to unwanted effects.

#### Borderline Personality Disorder

The efficacy and tolerability of DUL for treating symptomatic clusters of BPD has been evaluated in a 12-week, open-label trial (47) including a sample of 18 patients (14 completers: 9 females and 5 males, with 4 dropouts due to noncompliance) treated with DUL, 60 mg/day; no other psychotropic drug or psychological intervention was allowed during the trial. Psychodiagnostic evaluation included a semistructured clinical interview assessing frequency and severity of BPD symptoms (BPDSI), the CGI-S, and rating scales for depression (HDRS), anxiety (HARS), general psychopathology (BPRS), social and occupational functioning (Social Occupational Functioning Assessment Scale [SOFAS]), and somatization (Hopkins Symptom Checklist-90 Somatization Subscale [HSCL-90 SOM]). Results showed statistically significant improvements in three BPDSI items: impulsivity (*P* = 0.028), outbursts of anger (*P* = 0.0005), and affective instability (*P* = 0.001) and total score (*P* = 0.001), CGI-S mean score (*P* = 0.002), BPRS mean score (*P* = 0.001), HAM-D mean score (*P* = 0.035), SOFAS mean score (*P* = 0.0005), and HSCL-90 SOM mean score (*P* = 0.0005). DUL treatment was generally well tolerated, and the four dropouts were unrelated to tolerability. Most common adverse effects were nausea and headache, gastrointestinal symptoms, dizziness, and insomnia.

## Discussion

The prescription of antidepressants for psychiatric disorders other than MDD and GAD is a common practice. The evidence here reviewed suggests that DUL may be a potential treatment for treating different clusters of psychiatric symptoms.

Regarding the spectrum of mood disorders, the findings from the examined studies highlighted the efficacy and tolerability of DUL, with response rates ranging from 53% to >80%. DUL treatment significantly improved, with a satisfactory tolerability profile, dysthymia in adults (29), and pure DD of older adults (30), a form of dysthymia with typical onset in late life, characterized by the lack of psychiatric comorbidities and by a worse response to treatments (48). In PDD, DUL resulted effective in the short-term treatment (31) and for maintaining symptom improvement over time, with a positive impact on more general aspects of functioning and on social adjustment (32).

In SAD, promising results have been obtained from a single, small, open-label, short-term study (33); consequently, it is difficult to draw any conclusions about the real efficacy and safety of DUL in the longer term and in larger samples of SAD-affected patients. The available studies (34, 35) support evidence on the efficacy and tolerability of DUL as monotherapy in the treatment of PMDD, highlighting significant improvements in the core symptoms of irritability, affective lability, tension, depressed mood, and functional impairment; nevertheless, these results should be considered as preliminary, due to methodological limitations.

Concerning psychiatric conditions not belonging to the spectrum of mood disorders, uncertain results have been found. Positive results emerged for PD, schizophrenia, and borderline personality disorder. It seems almost intuitive that PD, being an anxiety disorder, can benefit from treatment with DUL, although the evidence comes from a single, open-label, and underpowered trial (38). In schizophrenia, results from two randomized, double-blind, placebo-controlled trials (42, 43) have documented the significant effect of DUL as add-on treatment on negative and general psychopathological symptoms. Antidepressant drugs are commonly used as an augmentation strategy to enhance the efficacy of antipsychotics (APs) (49, 50, 51), although it must be kept in mind that such strategy is not supported by robust scientific evidence regarding efficacy and safety and that it may substantially increase the risk of developing drug interactions and adverse events. In BPD, DUL improved several core symptoms of the disorder, such as anger outbursts, affective instability, and impulsivity; congruently with a possible effect also on the affective dimension, this finding indicates a potential use of DUL in at least a subgroup of BPD patients prominently exhibiting these clusters of features (47).

DUL resulted moderately effective in treating PTSD, with a response rate <50% (39); this result is too specific and scarcely generalizable, since it is referred to a special population (military veterans). However, it should be noticed that the disorder is characterized by generally high nonresponse rates across treatment strategies, which recommended medications vary across different guidelines and that both first- and second-line treatments mostly act by reducing expression of PTSD symptoms, rather than providing remission and relapse prevention (52).

Conflicting results emerged in OCD, eating disorders, and PE (36, 37, 38, 40, 44, 45). Possible reasons for these discrepancies in outcomes may derive from differences in study design and methodology, such as open-label, uncontrolled versus randomized, double-blind designs, and from differences in doses and treatment intervals. Furthermore, only few studies applied the ITT analysis for the data analyses, and the plausible effect of not accounting for dropouts with ITT data must be taken into account.

Findings from the double-blind, randomized, placebo-controlled trial in adult ADHD are quite difficult to interpret, since no statistically significant differences between DUL and placebo on primary measure for ADHD symptoms (CAARS-Inv : SV) were found; nevertheless, the authors stressed positive changes in general clinical and in secondary measures, concluding that DUL may represent a valuable treatment option in ADHD (46).

In all reviewed studies, DUL treatment was generally well tolerated; consistently through all trials, most reported adverse events were consistent with the known safety profile of duloxetine and included gastrointestinal disturbances, headache, sedation, dry mouth, sexual dysfunctions, sweating, and insomnia, mainly in the mild-to-moderate range. Nevertheless, the data showed that adverse effects and discontinuations due to adverse effects were not uncommon.

In terms of response and overall acceptability, and also due to the relative scarcity of head-to-head comparisons with other antidepressants, duloxetine has not been recommended as a routine first-line acute treatment for major depression (53). Nevertheless, the drug has been proven safe and effective for improving core emotional symptoms and general functioning in MDD (54, 55) and psychic and somatic anxiety symptoms in GAD (56, 57); in prospective studies of efficacy, duloxetine has been evaluated employing the remission concept (getting the patient asymptomatic), rather than the response concept (getting the patient 50% better), and with a focus on recovery, which includes both clinical and functional remissions.

## Conclusions

Duloxetine is a dual-acting agent with a well-established use in MDD and GAD and approved for several clinical conditions other than psychiatric disorders, such as urinary incontinence, neuropathic pain, and FM. Its most significant feature relies on the double way of action through the selective blockades of serotonin and NE reuptake in the central nervous system with scarce or lack of affinity for muscarinic, histamine 1 and β1 adrenergic receptors; therapeutic benefits are usually achieved at the dose of 60 mg/day, and the drug is generally well tolerated, with transient and/or minor adverse reactions.

Regarding the use of duloxetine in other psychiatric disorders, the evidence here reviewed suggests that duloxetine may be an effective treatment for mood spectrum disorders, with relatively high rates of response and remission, PD, several symptom clusters of borderline personality, and as add-on drug in schizophrenia with the aim of addressing negative symptoms. Modest, inconsistent, or conflicting results have been found for the potential efficacy of duloxetine in OCD, PTSD, eating, and sexual disorders. However, apart from sporadic randomized, double-blind, controlled studies, most of the evidence comes from several interesting open-label studies, although limited by short trial duration, small sample sizes, and by the lack of control groups; furthermore, the long-term efficacy of duloxetine has not yet been investigated. Information on the efficacy and safety of duloxetine in special populations such as adolescents and elderly patients and in potentially at-risk samples of subjects with impaired organ function is still limited. This relative paucity of data does not allow drawing firm conclusions on the potential role of duloxetine in the treatment of psychiatric disorders other than MDD and GAD; further randomized, placebo-controlled studies of adequate duration on larger samples are needed for better defining the whole therapeutic potential of this antidepressant.

## Author Contributions

MM, RZ, ES, and AB designed the study and supervised the methods and procedures, the various drafts, and the final version of the manuscript. PM and SL performed the initial search, independently reviewed and selected the references based on the inclusion and exclusion criteria, and clarified any disputes in the presence of a third expert reviewer (MM). GP, CC, and FB managed the literature searches and wrote the first drafts of the manuscript. All authors contributed to and have approved the final manuscript.

## Conflict of Interest

ES has previously received honoraria for speaking and consultation from AstraZeneca, Boehringer Ingelheim, Eli Lilly, Janssen, Lundbeck, and Pfizer.

The remaining authors declare that the research was conducted in the absence of any commercial or financial relationships that could be construed as a potential conflict of interest.
